# Evolving etiologies and rates of revision total knee arthroplasty: a 10-year institutional report

**DOI:** 10.1186/s42836-022-00134-7

**Published:** 2022-08-25

**Authors:** Matthew L. Brown, Pooya Javidan, Sam Early, William Bugbee

**Affiliations:** 1 Department of Orthopaedic Surgery, Cooper University Hospital, Sheridan Pavilion -3 Cooper Plaza, Suite 408, Camden, NJ 08103 USA; 2 Department of Orthopaedic Surgery, Kaiser Permanente, El Cajon, CA USA; 3 Shiley Center for Orthopaedic Research and Education, Scripps Clinic, La Jolla, CA USA; 4 Department of Orthopaedic Surgery, Scripps Clinic, La Jolla, CA USA

**Keywords:** Total knee arthroplasty, Revision, Failure, Etiology

## Abstract

**Background:**

The number of total knee arthroplasties (TKA) performed in the United States is projected to rise significantly, with a proportionate increase in the revision burden. Understanding the mechanism of failure in primary TKA is important as etiologies continue to evolve and reasons for revision change. The purpose of this study was to determine the reason for revision TKA at our institution among early and late failures and assess if the etiology has changed over a 10-year time-period.

**Methods:**

We identified 258 revision TKAs performed at our institution between 2005 and 2014. Reasons for revision TKA were categorized according to diagnosis. We also conducted subgroup analysis for TKA revisions performed within two years of the primary TKA (early failures) and those performed after two years (late failures). Revision TKAs were also grouped by year of primary TKA (before and after 2000) and time period in which the revision TKA was performed (2005–2009 and 2010–2014).

**Results:**

The most common reason for revision TKA was infection (29.3%), followed by aseptic loosening (19.7%), which together accounted for half of all revisions. Other indications for revision were instability (11.6%), osteolysis (10.4%), arthrofibrosis (8.1%), polyethylene (PE) wear (7.7%), malalignment/malposition (5.4%), patellar complication (3.1%), periprosthetic fracture (2.3%), pain (1.5%), and extensor mechanism deficiency (0.8%). Nearly half of early failures (47%) were due to infection. Osteolysis and PE wear made of a significantly higher proportion of revisions of TKAs performed prior to 2000 compared to index TKAs performed after 2000.

**Conclusion:**

At our institution, infection was the most common reason for revision TKA. Infection had a higher rate of early revisions. Proportion of TKAs revised for osteolysis and PE wear was higher for TKAs performed prior to 2000. Proportion of revision TKA for infection and instability were higher with TKAs performed after 2000.

## Introduction

Total knee arthroplasty (TKA) is one of the most successful surgeries currently performed in the United States [1]. The success of TKA, combined with an aging population and patients’ desire to remain active throughout retirement drives the growing demand for TKA. Projections estimate that demand for primary TKA to be almost 3.5 million annually in the United States by 2030 [2]. The number of revision TKAs will increase over time as a function of higher volume of primary TKA and the trend for primary knee arthroplasty being performed in younger, more active patients. The demand for revision TKA is projected at 268,200 cases by 2030 [2]. Revision TKA is associated with increased patient morbidity and mortality [3, 4], and imposes a significant financial burden on the health-care system [2, 5]. Understanding why primary TKAs fail may allow for improvements in patient selection and optimization, surgical techniques, and implant design. Although previous authors have investigated the etiology of TKA failure, the literature remains relatively sparse, especially regarding recent data from the United States. It is unclear if the surgical indication for revision TKA has remained similar despite regional differences and trends in practice patterns, and evolving technologies.

The primary purpose of this study was to determine the indications for revision TKA over a ten-year period at a single institution. Secondary goals included assessing if the etiology for TKA failure differed between: (1) early (less than 2 years) and late (greater than 2 years) failures, (2) primary TKAs performed prior to December 31, 1999 and those performed after January 1, 2000, and (3) revision TKA performed between January 1, 2005 through December 31, 2009 and those performed between January 1, 2010 through December 31, 2014. We hypothesized that reason for revision TKA would not change over time and that reason for revision at less than two years would be different compared to revision performed after two years and this would not change over time despite new techniques and technologies.

## Methods

The institutional total joint arthroplasty database was queried to identify all revision TKA cases performed over a ten-year period (January 1, 2005 – December 31, 2014) at our institution, which serves as a tertiary referral center for knee arthroplasty. This database includes prospectively collected data and is approved by our institutional review board (IRB). Exclusion criteria included revision of a previous unicompartmental knee arthroplasty (UKA) and any previously revised TKA. Failure mode was assumed to be the diagnosis listed by the attending surgeon in the operative note from revision TKA; the operative note and electronic medical record (EMR) were retrospectively reviewed to confirm the diagnosis listed in the operative note. TKA failure was categorized as: aseptic loosening, osteolysis, periprosthetic joint infection (PJI), instability, periprosthetic fracture, arthrofibrosis, polyethylene (PE) wear, patellar loosening, malalignment or malposition, extensor mechanism deficiency, or pain. As previous authors have suggested, etiology of failure can be multifactorial, and variability exists among surgeons in assigning failure mode. Furthermore, failure modes often exist along a continuum, especially aseptic loosening, osteolysis, and PE wear. Aseptic loosening was defined as prosthetic loosening in the absence of macroscopic PE wear. Osteolysis was defined as prosthetic loosening with macroscopic PE wear. PE wear was defined similar to previous authors as macroscopic polyethylene wear with stable, well-fixed implants [6–8]. PJI was diagnosed using criteria established by the Musculoskeletal Infection Society (MSIS), which have been periodically updated [9, 10]. Instability was diagnosed according to clinical examination and history as previously described [11]. If a secondary etiology for revision TKA was identified, only the primary etiology for revision was recorded. Failed TKAs were classified as either early or late using a definition similar to previous authors: early failures were defined as revision TKA performed less than two years from the index TKA [6, 7, 12]. Revision TKAs were stratified according to whether primary TKA was performed before or after January 1, 2000. January 1, 2000 was selected because this approximates the time period before and after improved polyethylene production and sterilization techniques were broadly adopted, which have been documented to impact PE wear rates [13]. Revision TKAs were also stratified according to when the revision TKA surgery was performed, *i*.*e*., January 1, 2005 through December 31, 2009 and January 1, 2010 through December 31, 2014. Time to revision and selected patient demographic details, including age, sex, and body mass index (BMI) were also recorded for each revision TKA.

### Statistical analysis

Descriptive statistics were calculated to summarize etiologies for revision TKA and time from index primary TKA to revision TKA. Chi-square tests were used to compare the etiology for revision TKA between time-period of both primary TKA and revision TKA. Time to revision between etiologies was compared using one-way ANOVA. SPSS version 13.0 was used for all analyses and an alpha level of 0.05 was used to determine statistical significance. An investigator with advanced training in statistics was involved in study design, data collection, and data analysis.

## Results

332 revision TKAs were performed at our institution from January 1, 2005 through December 31, 2014. 74 revision TKAs were excluded because the etiology was failed UKA or a previously revised TKA. 258 revision TKAs remained for analysis. 148 (57.5%) index primary TKAs had been performed at our institution and 110 (42.4%) had been performed at an outside institution.

**Fig. 1:**
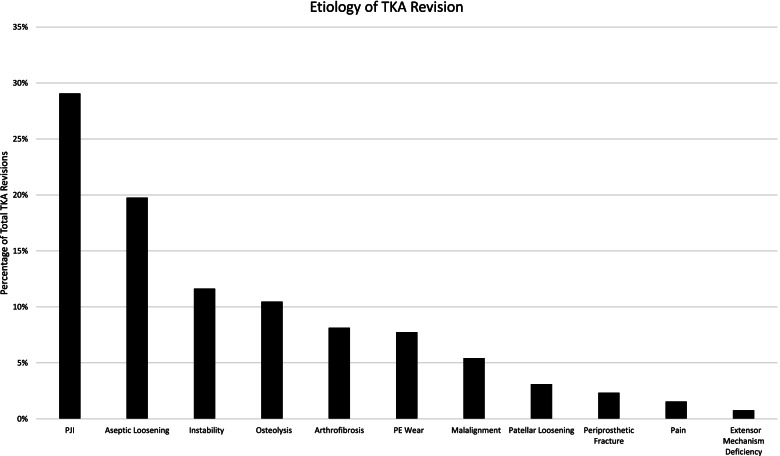
Etiology of TKA Revision. Percentage of total TKA revisions is displayed on the vertical axis and cause of revision is displayed on the horizontal axis

The most common etiology for revision TKA was PJI (*n* = 75, 29.3%), followed by aseptic loosening (*n* = 51, 19.7%), instability (*n* = 30, 11.6%), osteolysis (*n* = 27, 10.4%), arthrofibrosis (*n* = 21, 8.1%), PE wear (*n* = 20, 7.7%), malalignment (*n* = 14, 5.4%), patellar loosening (*n* = 8, 3.1%), periprosthetic fracture (*n* = 6, 2.3%), pain (*n* = 4, 1.5%), and extensor mechanism deficiency (*n* = 2, 0.8%) (Fig. 1). Selected patient characteristics, including sex, age at index TKA, and BMI are summarized in Table 1. There were no significant differences in terms of sex or BMI between causes for revision TKA. There was a significant difference in age between causes for revision TKA. Time to revision for aseptic loosening, osteolysis, and polyethylene wear, and periprosthetic fracture was longer compared to other etiologies (Table 1).

**Table 1:** Descriptive statistics for revision TKAs performed during the study period, including select patient demographics, details regarding the primary TKA and revision TKA. Percentages listed for time to revision, primary time period, and revision time period are from the total number of revisions included in the given time period. Percentages for revision description are percent of total for given etiology. Data listed for BMI, age at index TKA, and time to revision TKA are mean ± standard deviation

	**All Causes**	**PJI**	**Aseptic Loosening**	** Instability**	** Osteolysis**	**Arthrofibrosis**	** PE Wear**	** Malalignment**	**Patellar Loosening**	**Periprosthetic Fracture**	** Pain**	** Extensor Mechanism Deficiency**
**Revision TKAs**	258	75 (29.3%)	51 (19.7%)	30 (11.6%)	27 (10.4%)	21 (8.1%)	20 (7.7%)	14 (5.4%)	8 (3.1%)	6 (2.3%)	4 (1.5%)	2 (0.8%)
**Male**	120	43	21	10	13	8	11	8	5	0	0	1
**BMI [Kg/m**^**2**^**]**	29.3	28.6 +/- 6.2	29.5 +/- 5.1	31.7 +/- 7.4	28.0 +/- 3.9	29.3 +/- 5.9	30.1 +/- 6.4	28.5 +/- 4.6	30.7 +/- 6.5		25.5 +/- 5.9	32.0
**Age at Index TKA [years]**	62.7 +/- 10.7	67.2 +/- 11.7	62.7 +/- 9.4	59.8 +/- 11.9	56.9 +/- 8.8	64.3 +/- 7.9	55.8 +/- 11.1	61.9 +/- 7.1	61.9 +/- 9.4	67.3 +/- 6.6	61.2 +/- 4.7	55.1
**Time to Revision TKA [years]**	6.8 +/- 6.3	4.4 +/- 5.1	8.4 +/- 5.8	4.4 +/- 5.3	15.7 +/- 3.9	3.2 +/- 2.6	11.0 +/- 5.7	3.8 +/- 3.6	6.6 +/- 5.5	11.4 +/- 9.4	3.0 +/- 4.4	2.2
**Time to Revision:**												
<2 years	76 (29.3%)	36 (47.4%)	4 (5.3%)	14 (18.4%)	0	10 (13.2%)	1 (1.3%)	6 (7.9%)	2 (2.6%)	0	3 (3.9%)	0
≥2 years	182 (70.7%)	39 (21.9%)	47 (25.7%)	16 (8.7%)	27 (14.8%)	11 (6.0%)	19 (10.4%)	8 (4.4%)	6 (3.3%)	6 (3.3%)	1 (0.5%)	2 (1.1%)
**Primary Time Period:**												
Before 2000	76 (29.3%)	10 (13.2%)	15 (19.7%)	3 (3.9%)	25 (32.9%)	1 (1.3%)	14 (18.4%)	1 (1.3%)	0	5 (6.6%)	0	1 (1.3%)
After 2000	182 (70.7%)	65 (35.7%)	36 (19.8%)	27 (14.8%)	2 (1.1%)	20 (11.0%)	6 (3.3%)	13 (7.1%)	7 (3.8%)	1 (0.5%)	4 (2.2%)	1 (0.5%)
**Revision Time Period:**												
2005-2009	109 (38.2%)	35 (32.1%)	14 (12.8%)	12 (11.0%)	10 (9.2%)	9 (8.3%)	14 (12.8%)	5 (4.6%)	3 (2.8%)	4 (3.7%)	2 (1.8%)	1 (0.9%)
2010-2014	149 (57.8%)	40 (27.3%)	37 (24.7%)	18 (12.0%)	17 (11.3%)	12 (8.0%)	6 (4.0%)	9 (6.0%)	5 (3.3%)	2 (1.3%)	2 (1.3%)	1 (0.6%)
**Revision Description:**												
All components	151	45 (60%)	44 (84.6%)	8 (26.7%)	27 (100%)	9 (42.9%)	0	8 (57.1%)	0	6 (100%)	3 (75%)	1 (50%)
PE exchange	83	30 (40%)	0	22 (73.3%)	0	11 (52.4%)	20 (100%)	0	0	0	0	0
Femoral component	2	0	2 (3.8%)	0	0	0	0	0	0	0	0	0
Tibial component	5	0	3 (5.8%)	0	0	0	0	1 (7.1%)	0	0	1 (25%)	0
Patellar component	12	0	0	0	0	1 (4.8%)	0	3 (21.3%)	8 (100%)	0	0	1 (50%)
Unknown	5	0	3 (5.8%)	0	0	0	0	2 (14.2%)	0	0	0	0

*Abbreviations*: *PJI* periprosthetic joint infection, *BMI* body mass index, *PE* polyethetlene, *TKA* total knee arthroplasty

**Fig. 2:**
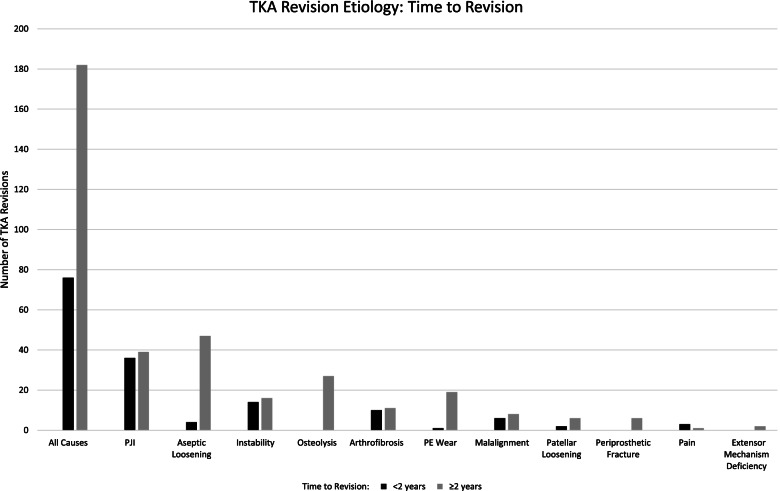
TKA Revision Etiology: Time to Revision. Number of TKA revisions is displayed on the vertical axis and cause for revision is displayed on the horizontal axis. Black bars indicate TKA revision performed less than two years after primary TKA. Grey bars indicate TKA revision performed more than two years after primary TKA

75 (29.3%) revision TKAs were performed in the early period (< 2 years from index TKA) and 183 (70.7%) were done in the late period (≥ 2 years from the index TKA) (Fig. 2). Patient age at surgery was significantly higher for early failures (66.4 years *vs*. 61.4 years, *P* < 0.001). Rate of revision TKA was significantly increased for PJI (*P* < 0.001) and instability (*P* < 0.05) among early failures. Conversely, rate of revision TKA was significantly decreased for aseptic loosening (*P* < 0.001), osteolysis (*P* < 0.001), and PE wear (*P* < 0.001) among early failures.

**Fig. 3:**
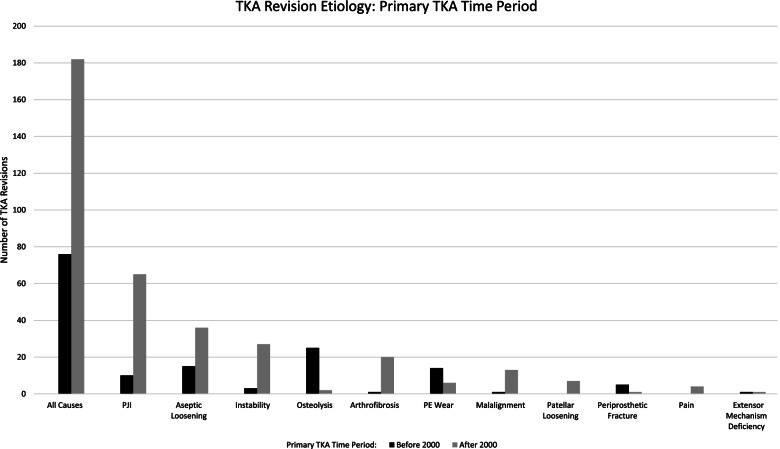
TKA Revision Etiology: Primary TKA Time Period. Number of TKA revisions is displayed on the vertical axis and the cause for revision is displayed on the horizontal axis. Black bars indicate primary TKA performed before January 31, 1999. Grey bars indicate primary TKA performed after January 31, 1999

Revision TKA was performed for 76 (29.3%) index TKAs implanted prior to 2000 and 182 (70.7%) performed after 2000 (Fig. 3). Patient demographics were not statistically different between the two periods in terms of sex or BMI, however, patient age was significantly greater in the group undergoing index TKA after 2000 (64.3 years *vs*. 59.3 years, *P* < 0.001) (Table 1). There was a significant increase in rate of revision TKA for PJI (35.7% *vs*. 13.2%, *P* < 0.001), instability (14.8% *vs*. 3.9%, *P* < 0.05), and arthrofibrosis (11.0% *vs*. 1.3%, *P* < 0.01) for TKAs performed after 2000. Conversely, the rate of revision TKA was significantly decreased for osteolysis (1.1% *vs*. 32.9%, *P* < 0.001), and PE wear (3.3% *vs*. 18.4%, *P* < 0.001) for TKAs performed after 2000.

**Fig. 4:**
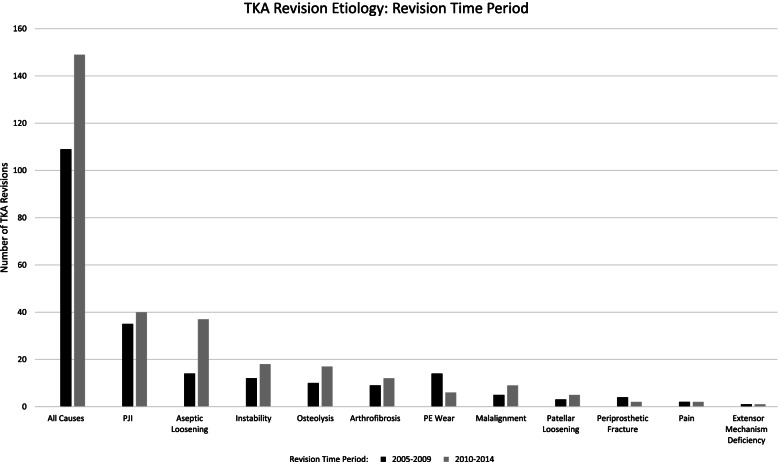
TKA Revision Etiology: Revision Time Period. Number of TKA revisions is displayed on the vertical axis and the cause for revision is displayed on the horizontal axis. Black bars indicate revision TKA performed between 2005–2009. Grey bars indicate revision TKA performed between 2010–2014

109 (38.2%) revision TKAs were performed between 2005–2009 while 149 (57.8%) were performed between 2010–2014 (Fig. 4). Patient demographics were not statistically different between the two periods (Table 1). Rate of revision TKA was significantly increased for aseptic loosening (12.8% *vs*. 24.7%, *P* < 0.05) from 2005–2009 compared to 2010–2014. Conversely, there was a significantly decreased rate of revision for PE wear (12.8% *vs*. 4.0%, *P* < 0.01) from 2005–2009 compared to 2010–2014.

The majority of patients had all components revised. This was done in 151 (58.5%) cases. Polyethylene exchange was the next commonest procedure, which was done in 83 (32.2%) cases. Isolated component revision was performed in 19 cases, with femur only in 2, tibia only in 5, and patella only in 12. Status of components was unknown for 5 cases. Details of components exchanged for each diagnosis are provided in Table 1.

## Discussion

Demand for revision TKA is on the rise. This increase is likely multifactorial: (1) primary TKA is being performed more frequently and is more accepted by younger, more active patients, (2) a better understanding of and ability to diagnose TKA failure modes, and (3) improved surgical techniques to address failed TKA. Revision TKA is economically costly, with a total yearly expenditure of $13 billion projected by 2030 in the US [14], and imposes significant morbidity and mortality on patients [3]. As the US healthcare system appropriately continues to emphasize value, understanding the etiology of revision TKA has become particularly important. Previous investigators have reported on revision TKA and selected results are summarized in Table 2. Registry and national databases offer a broad overview of etiologies for revision TKA but the lack of granularity precludes detailed analysis of cases to best determine the etiology of failure and also prevents analysis of patient level data. Retrospective reviews allow for more in-depth analysis but are often limited to relatively small numbers and to certain geographic areas and time periods and are restricted by other methodologic flaws inherent to retrospective studies. Our current study provides additional data regarding revision TKA at a tertiary arthroplasty referral center within the United States and supports and reinforces similar previous studies in the literature.

**Table 2:** Summary of selected publications regarding revision TKA

**Author**	**Design**	**Time Period**	**Region**	**Revision TKAs**	**Early vs Late**	**Infection**	**Aseptic Loosening**	**Instability**	**Osteolysis**	**Periprosthetic Fracture**	**Polyethylene Wear**	**Malalignment**	**Dislocation**	**Patellar Complications**	**Extensor Mechanism Failure**	**Implant Failure**	**Arthrofibrosis**	**Pain**	**Other**	**Disease Progression**
Lewis 2020	Registry	2003-2017	Sweden, Australia, USA	78,151	-	21,030 (26.9%)	21,808 (27.9%)	5,407 (6.8%)		1,732 (2.2%)	5,307 (6.8%)			6,234 (8.0%)		1,409 (1.8%)	1,761 (2.3%)	4,148 (5.3%)	4,590 (5.9%)	4,707 (6.0%)
Pietrzak 2019	Retrospective review	2013-2016	France	255	Early: 96 (37.6%) Late: 159 (62.4%)		85 (33.3%)	39 (15.3%)			8 (3.1%)			24 (9.4%)	10 (3.9%)	14 (5.5%)	70 (27.5%)	1 (0.4%)	4 (1.6%)	
Postler 2018	Retrospective review	2010-2015	Germany	402	Early: 106 (26.3%) Late: 207 (73.7%)	146 (36.3%)	87 (21.6%)	27 (6.7%)		55 (13.7%)	21 (5.2%)				15 (3.7%)	8 (2.0%)	18 (4.5%)	24 (6.0%)		
Boelch 2018	Retrospective review	2008-2016	Germany	1,143	-	344 (30.0%)	178 (15.6%)	193 (16.9%)		41 (3.6%)	54 (4.7%)	70 (6.1%)		108 (9.4%)	31 (2.7%)	16 (1.4%)	49 (4.3%)		58 (5.1%)	
Delanois 2017	Database	2009-2013	USA	337,597	-	88,662 (20.4%)	88,327 (20.3%)		11,118 (2.6%)	6,032 (1.4%)	13,897 (3.2%)		32,708 (7.5%)			12,905 (3.0%)			100,872 (23.2%)	
Lee 2017	Retrospective review	2003-2012	Korea	206	-	120 (58.3%)	25 (12.1%)	11 (5.3%)		36 (17.5%)	13 (6.3%)	1 (0.4%)								
Thiele 2015	Retrospective review	2005-2010	Germany	358	Early (<1y): 71 (22%) Mid (1-3y): 163 (42%) Late (>3y): 124 (36%)	52 (14.5%)	78 (21.8%)	78 (21.8%)		12 (3.3%)	25 (7.0%)	74 (20.7%)		21 (5.9%)	2 (0.6%)		16 (4.5%)			
Sharkey 2014	Retrospective review	2003-2012	USA	781	Early (<2y): 294 (37.6%) Late (>2y): 487 (62.4%)	214 (27.4%)	312 (39.9%)	68 (8.6%)	-	37 (4.7%)	27 (3.5%)	16 (2.0%)	-	54 (7.0%)	2 (0.3%)	-	35 (4.5%)	-	-	-
Kasahara 2013	Retrospective review	2006-2011	Japan	140	-	33 (24%)	56 (40%)	13 (9%)	13 (9%)	5 (4%)						9 (6%)			11 (8%)	
Hossaini 2010	Retrospective review	1999-2008	United Kingdom	349	Early: 112 (32.1%) Late: 237 (67.9%)	114 (32.7%)	52 (14.9%)	27 (7.7%)	-	29 (8.3%)	43 (12.3%)	23 (6.6%)	-	15 (4.3%)	5 (1.4%)	3 (0.9%)	9 (2.6%)	-	-	29 (8.3%)
Bozic 2010	Database	2005-2006	USA	60,436	-	15,233 (25.2%)	9,711 (16.1%)	-	1,910 (3.2%)	900 (1.5%)	2,967 (4.9%)	-	4,268 (7.1%)	-	-	5,852 (9.7%)	-	-	9,287 (15.4%)	
Sharkey 2002	Retrospective review	1997-2000	USA	212	Early (<2y): 55.6% Late (>2y): 42.4%	37 (17.5%)	51 (24.1%)	45 (21.2%)	-	6 (2.8%)	53 (25.0%)	25 (11.8%)	-	11 (5.1%)	14 (6.6%)	-	31 (14.6%)	-	-	-
Fehring 2001	Retrospective review	1986-1999	USA	440	Early (<5 yrs): 279 (63%)	105 (38%)	45 (16%)	74 (27%)			21 (7%)			22 (8%)					12 (5%)	

PJI was the most common etiology for revision in our study, accounting for 29.3% of revision TKAs overall and nearly half (47.4%) of early revisions. PJI being the commonest etiology for revision TKA is similar to the results reported by other authors from large database studies [15, 16], and many retrospective reviews [7, 12, 17–19]. PJI was not the most frequent reason for revision TKA in one registry study [20] and other retrospective reviews [6, 8, 21, 22]. There did not appear to be any chronological trend in the literature for the proportion of revision TKAs performed for PJI. Despite significant work to identify risk factors for PJI and implementation of preoperative patient optimization protocols to minimize the risk of PJI [23–27], PJI remains a significant mode of failure and requires further effort within the arthroplasty community.

Aseptic loosening was the next most prevalent etiology for revision TKA in our study, accounting for 19.7% of revisions. Aseptic loosening was more common among late revisions (25.7%) compared to early revisions (5.3%). Aseptic revision was a common cause for revision in the literature, with percentage of revision due to aseptic loosening ranging from 14.9% to 40.0% and was reported as the most frequent etiology of revision by several investigators [6, 20–22]. Sharkey *et al*. commented that cementless fixation was a risk factor for aseptic loosening. Previous cementless knee designs had documented high failure rates, more recent cementless designs have shown equivalent survivorship at short- to mid-term followups [28–31]. Cement viscosity has also been investigated as a source of loosening [32–34]. Our study was not designed to investigate the differences in cemented *vs*. cementless fixation or cement viscosity. However, we believe these issues require further investigation to determine their impact on implant fixation.

Instability accounted for 11.6% of revisions in our study. Instability was more common among patients undergoing early revision (18.4% *vs*. 8.7%) and among patients with primary TKA performed after 2000 (14.8% *vs*. 3.9%). Revision rates for instability reported in the literature ranged from 5.3%–21.8%. Sharkey *et al*. reported lower incidence of instability over time [6, 21], perhaps due to improved implant designs and surgical techniques. Instability was unchanged between revisions performed 2005–2009 and 2010–2014 in our study. However, our finding of increased rate of revision for instability among primary TKAs performed after 2000 is counterintuitive as conventional thinking is that improved implant design will translate to improved stability. However, one explanation for this seemingly counterintuitive increase in revision for instability is an improved understanding of the problem and its presentation with concomitant improvement in surgical techniques to address instability. Another possible explanation is that newer implant designs failed to improve knee kinematics and stability compared to previous implants. It is beyond the scope of the current paper to explain this observation and further work is required.

Osteolysis accounted for 10.4% of revisions in our study. All revisions for osteolysis were performed more than 2 years from index TKA and the vast majority (25/27) were performed in primary TKAs performed prior to 2000. These data correspond to improved understanding of PE failure modes and the transition to sterilization of PE in inert environment free from oxygen. We do not, however, have information on PE used in TKAs that failed for osteolysis and we cannot definitively link the decreased rate of failure secondary to PE wear to the improvement in PE sterilization. Osteolysis was reported as failure mode ranging from 2.6–9% in the literature, however, only 3 studies listed osteolysis as a failure mode, which is likely due to overlap between osteolysis and PE wear when assigning failure modes.

Arthrofibrosis accounted for 8.1% of revisions in our study, including 13% of early revisions and 6% of late revisions. Arthrofibrosis, as a cause for revision, ranged from 2.3–27.5% in the literature. Arthrofibrosis is a multifactorial process and the diagnosis and treatment of this pathology are somewhat variable, which makes comparison of results difficult.

PE wear accounted for 7.7% of revisions in our study and 19 of 20 failures occurred greater than 2 years, as would be expected. 14 of 20 failures were in primary TKAs implanted prior to 2000, which, we again hypothesize, is related to improved methods of PE sterilization. Revision for PE wear reported in the literature ranged from 3.1–25.0%. There appears to be a trend in the published literature for decreased rates of failure attributed to PE wear over time.

Malalignment accounted for 5.4% of revisions in our study. The rate of revision for malalignment in the literature ranges from 0.4–20.7%. The understanding of and ability to analyze component position using CT protocols has increased over time.

Patellar loosening, periprosthetic fracture, and pain each accounted for less than 10 revisions in our series.

The spectrum and timing of etiologies of revision TKA in this study were similar to previously published literature and show that early revisions are rarely implant-related but more related to patient and surgeon technical factors, such as infection, arthrofibrosis, instability and malalignment. Early revision of a primary TKA is a devastating complication for patient and surgeon alike. Focusing on causes and prevention of early revision, such as modification of patient-related variables and adoption of technologies such as navigation and robotics to allow for more precise and accurate prosthesis implantation may prove valuable. However, at present, these technologies have demonstrated improved component positioning but no change in revision rates. Nonetheless, this study, while unable to identify changes in incidence, suggests that significant changes in etiology have not occurred.

Late revisions were more consistently associated with implant-related causes, such as loosening, wear and osteolysis. This is not unexpected, but the data confirm the biggest risk to long-term TKA success remains implant-related variables. Advances in technology, such as enhanced polyethylene, improved cementing techniques, modern cementless design may ultimately reduce some late revisions, but this was not demonstrated in our study. We were surprised by the relatively low incidence of periprosthetic fracture as a reason for revision, but did not study the rate of revision *vs*. ORIF and prosthesis retention in our population.

This study has several limitations. First, our study is limited by variability inherent to determining the etiology of TKA failure. As previous investigators have suggested, failure is often multifactorial and modes of failure may overlap, particularly aseptic loosening, osteolysis, and PE wear. Second, our study design likely systematically underreported periprosthetic fracture and extensor mechanism deficiency as failure modes following primary TKA because we only identified periprosthetic fracture or extensor mechanism deficiency as etiology of failed TKA if the problem required revision TKA. Periprosthetic fracture and extensor mechanism disruption are often amenable to operative fixation or repair without revision TKA. Third, we do not have details on the implants used in the primary TKA and we cannot comment on specific implant designs or fixation mechanisms as being associated with higher revision rates.

## Conclusion

Our data demonstrated that PJI remains the dominant failure mode, especially for early failures. Aseptic loosening, instability, and arthrofibrosis are also prevalent modes of failure. Surgeons should exercise care with patient selection and optimization efforts prior to TKA to minimize risk of PJI. Aseptic loosening may be related to implant design, cement viscosity, and fixation type (cementless *vs*. cemented) and continued investigation of these issues to maximize long-term fixation are merited. Instability is largely dependent on surgical technique implant design and, with better understanding of this failure mode, there may be an initial increase in the revision burden due to heightened awareness.

## Data Availability

The datasets analyzed during the current study are available from the corresponding author on reasonable request.

## Abbreviations

TKA: Total knee arthroplasty
PE: Polyethylene
IRB: Institutional review board
EMR: Electronic medical record
PJI: Periprosthetic joint infection
MSIS: Musculoskeletal Infection Society
